# The First Record of an Aggressive Form of Ocular Tumour Enhanced by Marek's Disease Virus Infection in Layer Flock in Al-Najaf, Iraq

**DOI:** 10.1155/2024/1793189

**Published:** 2024-09-30

**Authors:** Aoula Al-Zebeeby, Ali Hadi Abbas, Haider Abas Alsaegh, Furkan Sabbar Alaraji

**Affiliations:** ^1^ Department of Pathology and Poultry Diseases Faculty of Veterinary Medicine University of Kufa, Al-Najaf Al-Ashraf, Kufa, Iraq; ^2^ Department of Veterinary Microbiology Faculty of Veterinary Medicine University of Kufa, Al-Najaf Al-Ashraf, Kufa, Iraq

## Abstract

Marek's disease (MD) is a highly infectious poultry illness with a tendency to form tumours in peripheral nerves and internal organs of affected birds. Tumours accompany MD, mostly caused by oncogenic Gallid alpha herpesvirus 2 (MD Herpes virus serotype I). Studies on avian tumours associated with MD infection are limited in Iraq. In the presented study, the positive samples of ocular tumour were 168 out of 282 MD positive samples, which accomplished in farm suffered from an unexpectedly high mortality rate. We investigated a rapidly developed tumour mass that was observed in an MD-vaccinated layer flock that showed obvious clinical signs of MD, accompanied by forming a small lump in one eye at age 21 weeks, which developed to a big lump at week 28 of age, leading to death. The diagnosis MD infection was confirmed by a Polymerase Chain Reaction (PCR) amplification of a specific region of the target gene *meq* of the causative agent, followed by Sanger sequencing and BLASTn search of the sequence against the NCBI nucleic acid database, resulted in Gallid alpha herpes virus 2 strain, and according to the phylogenetic analysis, the sequence from this study was uniquely clustered in its own branch in the tree. Histopathological examination of the ocular tumour core revealed aggregation of neoplastic cells and haemorrhage that replaced the normal eye tissue, as well as early tumour formation in internal organs such as the lung and liver. In addition, abnormal lesions are susceptible to tumours in the gizzard and spleen. To our knowledge, this is the first record of an aggressive MD virus infection-mediated ocular tumour in a layer flock in Al-Najaf province, Iraq.

## 1. Introduction

Marek's disease is one of the most contagious, widespread lymphoproliferative diseases in poultry which is characterised by a rapid onset of lymphoid tumours in internal organs and nervous tissue, resulting in high economic losses in the poultry industry [1, 2]. MD, among other poultry diseases, has the highest economic impact on poultry production worldwide [3, 4]. The economic losses occur due to MD, starting from the first episodes of production to the very end of the production cycle [5–7].

Vaccinations have drastically minimised the chance of an incidence of such an MD infection. However, vaccinated chickens are susceptible to MD infection with MD virus strains that are shed in the field's environment [8]. This issue may lead to the evolution of the virus into a more virulent one [9, 10]. MD is caused by three serotypes of alpha-Herpes virus; serotype I is considered as an oncogenic one, while the other two serotypes are not oncogenic [11, 12].

The infection caused by the oncogenic serotype is characterised by a number of phases, namely, an early infection cytolytic phase (two to seven days postinfection), a latent phase (seven to ten days postinfection), a late cytolytic and permanent immune-suppression phase (eighteen days postinfection onwards), and a transformation and proliferation phase of lymphocytes type T with tumour development (three to four weeks postinfection onwards) [13]. Basically, the main mechanism of MDV oncogenicity occurs through the transformation of T-lymphocytes, resulting in tumour initiation and development in addition to immunosuppression [14]. It has been mentioned that B cells are dispensable completely in the case of MDV pathogenesis and tumour initiation. In the B cell absence, MD virus can easily replicate in T cells, namely, CD^8+^ and CD^4+^ [4, 15]. Recently, a study revealed that the MD virus replication occurs in other lymphocytes, such as natural killer cells [16]. In addition to the ability of the MD virus to replicate in the T cells, it can also create a latency in the T cells [17]. In latently infected cells and tumour formation, MD virus inserts its genome into the host chromosome at the telomere's region [18]. This viral genome insertion is vital for T cell transformation. Rapidly, T cell lymphomas' formation is the most distinctive feature of MD virus infection [19].

MD is a progressive disease with different signs, which has four overlapping forms including a characteristic neurolymphomatosis (classical form of MD), acute form, cutaneous form, and ocular lymphomatosis. The tumours that accompany this infection occur in different organs and tissues like the lung, liver, sciatic nerve, proventriculus, feather follicle, spleen, and ovary [14, 20]. The ocular form of MD associated with different pathological lesions according to the anatomical site of infection. Mostly, such lesions are resulting in blindness leading to death due to starvation [21]. In the primary lesions of the ocular form are characterised by hypertrophy of endothelial cells, infiltration of lymphocytes (mainly CD^8+^), heterophils, plasma cells, and macrophages in different eye tissues including the iris, ciliary layer, and choroid layer. While the late lesions' lymphohistiocytic uveitis, pectenitis, keratitis, retinitis, vitreitis, and diffuse retinal necrosis. In this case, the inflammatory cells infiltrated to the affected area such as plasma cells, granulocytes, macrophages, and the infected T cell CD^8+^ and CD^4+^ in different sizes [21, 22].

Generally, the method used for MD diagnosis is based on clinical signs and gross appearance [23]. However, the clinical signs are not always noticeable, for instance, the decrease in the growth rate and egg production [2]. Therefore, the histopathological examination of the isolated lesions is critical for MD diagnosis. Such examination would describe the main precisely changes that occur in the tissue accompanied tumour formation. Especially in the cases of MD virus mediated-tumour proliferation (lymphomatous) lesions, which consist of accumulation of the infected lymphocytes [24]. In addition, molecular-based methods like Polymerase Chain Reaction (PCR) of the causative agent are one of the best methods to confirm the diagnosis of the disease [18, 19].

It has been reported that several genes have an important role in MD pathogenicity. One of these genes, Marek's EcoRI-Q (*meq*) gene, has a crucial role in MD virus oncogenicity. The expression level of *meq* correlates with increased susceptibility to form tumours in MD-infected birds. Therefore, this gene is considered one of the best targets for MD virus diagnosis [25–28].

In the present study, we investigated Marek's disease outbreak in a vaccinated layer flock and recorded for the first time unprecedented aggressive form of ocular tumour accompanied by the early formation of visceral tumours following this viral infection.

## 2. Materials and Methods

### 2.1. Samples

168 samples were collected from a vaccinated layer flock (Lohmann Brown Classic Chicks) that suffered from an MD outbreak and an increase in the mortality rate. After post mortem and gross examination, the affected tissues including the tumour core of the eye, advance front tumour, and visceral samples were washed with PBS and then fixed with 10% formalin (Chemanol, KSA) for (24 hours). Later, the formalin was changed and kept for another (24 hours) for further investigation. The study was conducted from January till April, 2024. All the collected samples were located at Agricultural Middle East Company for Animal Production in Al-Najaf Province, Iraq.

### 2.2. Ethical Approval

The current study was approved by the institutional animal care and use committee/University of Kufa as documented at the official request no. 1203 on 07th of February 2024.

### 2.3. Histopathological Examination

After the postmortem examination took place, the whole tumour eye was collected. While other organ samples were collected, a piece measuring 5 cm was excised from the affected tissue. Then, several steps were performed for preparing samples, such as PBS washing, the 10% formalin fixation step (Chemanol, KSA), and storing them in clean containers. 24 hours later, the ocular tumour was cut into a transverse section, and all samples, including the ocular tumour, were fixed with freshly prepared 10% formalin for another 24 hours. After the fixation step, the dehydration process was done by using a series of increasing concentrations of ethanol (Retouch, China). Then, the tissue samples have been paraffin embedded (Chemact Petrochemicals, China), sectioned, mounted on the slide, and stained with haematoxylin and eosin stain (Leica Biosystems, USA). Finally, histopathological changes were detected using a light microscope [29].

### 2.4. DNA Extraction and Marek's Disease Detection Using PCR

DNA extraction was done from tumour eye tissue samples using Cells and Tissue DNA Isolation Kit/Norgen kit (Cat. No. 53100) (Norgen Biotek, Canada) according to the manufactured protocol. Then, the extracted DNA was stored at −80°C for subsequent PCR detection. The *Meq* gene was targeted by a primer pair F GAATCTTCCCTGCATTGTGTC, R ATCTGGCCCGAATACAAGGAA with expected product size (196 pb) [30]. The thermocycler settings for DNA amplification were as follows: denaturation 94°C for 30 seconds, annealing 51°C for 30 seconds, extension 72°C for 1 seconds, final extension 72°C for 5 min, and final hold 4°C. DNA products were then subject to electrophoresis in 0.8% agarose gel, for 45 minutes at 75 volts. The PCR product was sent to MACROGEN® for Sanger sequencing, and then the sequence was analysed and quality trimmed using SnapGene 5.2.4 software and then submitted to NCBI BLASTn search against the nonredundant nucleic acid database using default settings of NCBI Blast.

Sequence data: partial DNA sequence of *meq* gene has been submitted to NCBI under an accession number PP551947, isolate MDV/Najaf/23 (146 bp): TAAAGCCTCTCCGGCTTCCGGGAGCCGGTTGGTAGGGGAGACAGGGTAAAAAGGGGAGCCTGGCCAACAGGACAAAGCTGAGCGTAAACCGTCCCCGGCGATGGAGGGGTACACTCCTCGGTAACAGGACACAATGCAGGGAAGAT.

### 2.5. Phylogenetic Analyses

17 were selected randomly from the BLASTn results sequences Supplementary file 1. To retrieve *meq* gene samples from Iraq deposited in GenBank, keyword search was undertaken with phrase “Gallid alphaherpesvirus 2 + *meq* gene Iraq” 9 sequences were selected randomly and DNA sequences were stored in Supplementary file 2. Then, DNA sequence files were merged in one FASTA format file for the alignment Supplementary file 3. For further reference, the accession numbers of all sequence used in the analysis are stored in Supplementary file 4. DNA sequence alignment was achieved using MUSCLE alignment algorithms [31] implemented in MEGA X software version 10 [32], and then the alignment was edited manually, and columns with 10% gaps were removed; this was resulted in an alignment with no gaps, which then stored in Supplementary file 5, that used as an input to constructing a phylogenetic tree. MEGA X software was used to generate a neighbour joining tree of DNA sequences with the Jukes-Cantor evolutionary model with bootstrapping of 500 to generate a NWK file format with tree information (Supplementary file 6).

## 3. Results

### 3.1. Suspected Marek's Disease Enhances Ocular Tumour Progression

**Figure 1:**
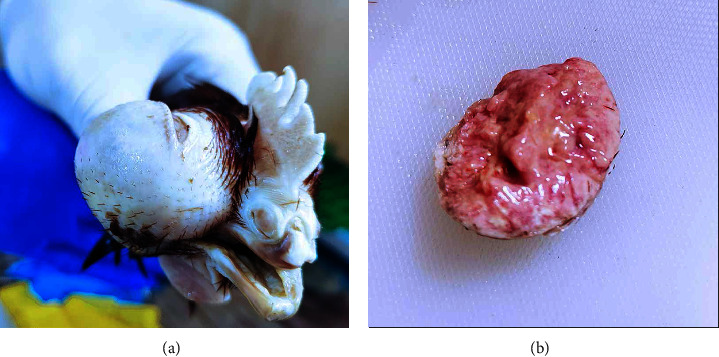
Gross lesion of bird eye under age 28 weeks: (a) large lump replaced the normal eye; (b) eye tumour core.

In order to investigate the main cause of the lesion and the death that was seen in the infected birds, the case history of the infected birds was recorded as these birds were vaccinated with the MD vaccine of one-day old. The infection started in chicks under the age of week 21 as a small lump in one eye accompanied by general clinical signs of MD, including depression, a decrease in growth rate, and egg production. Then, the lump continued to increase in size; by week 28 of the birds' age, most of the infected birds were found dead. The gross examination took place, which showed a very large mass that replaced the normal eye (Figures 1(a) and 1(b)).

### 3.2. Molecular Confirmation and Phylogenetic Analysis

**Figure 2:**
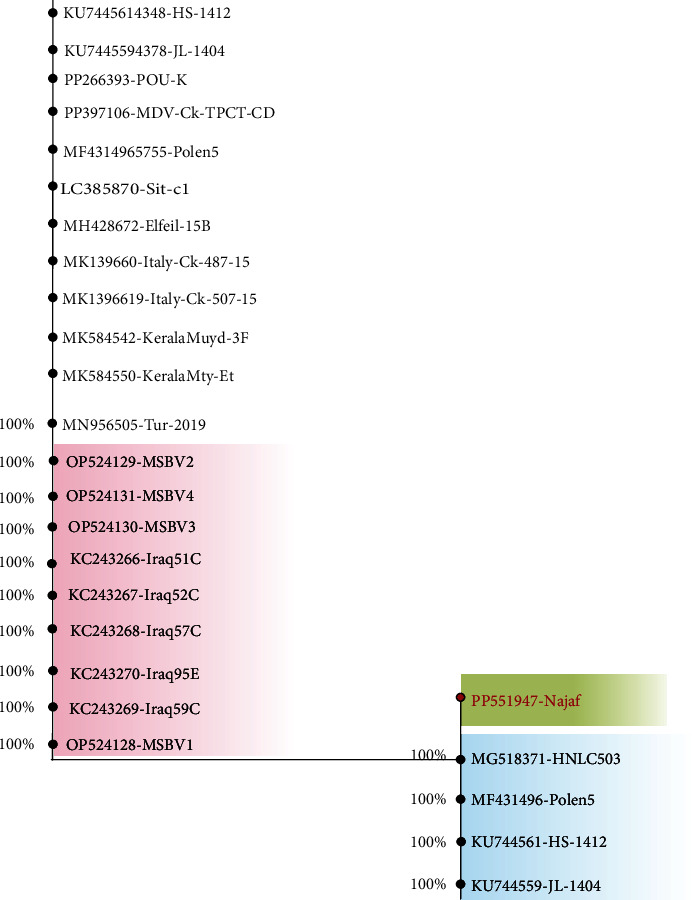
Phylogenetic tree of partial *meq* gene sequence. The sequence presented in this study (red text colour with gradient green shading) separated uniquely in one branch with 100% bootstrap confidence, the closest clade is formed by sequences mostly from China (gradient blue coloured shade). However, sequences from Iraq (gradient red colour shade) with all other sequences are in one clade. The tree constructed using MEGA X software using neighbour joining method with Jukes–Cantor DNA model testing 56 DNA shared loci.

The PCR results revealed that all eye tumour samples were positive. The PCR products indicated a single band collected from agarose gel (Supplementary Figure S1). To confirm the MD diagnosis, the PCR yield of 196 base pairs (bp) of 30 samples out of 163 was sequenced. The DNA sequence search results showed all sequences with a sequence match of 93.84% of *meq* gene belonging to Gallid alpha Herpes virus 2 isolated. It is worth mentioning that no sequences from Iraq were shown by this initial analysis. However, phylogenetic analysis of selected sequences from BLASTn search with sequences of samples isolated from Iraq revealed that the sequence from this study stud out in a monophyletic branch leaving the rest of sequences clustered in two separated branches with sequence from China and Poland formed the closest group. Interestingly, other sequences from Iraq clustered in the biggest collection of sequences but clustered away from the sequence of this study (Figure 2).

### 3.3. Histopathological Detection of MD Virus-Mediated Ocular Tumour

**Figure 3:**
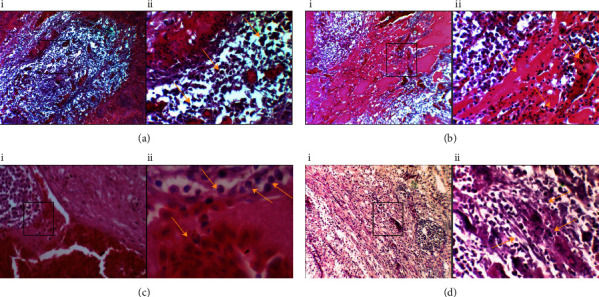
Histopathological sections of the ocular tumour mass in a Marek's disease-infected bird. (a) (i and ii) ocular tumour core section characterised by monomorphic neoplastic cells (arrows). (b) (i and ii) ocular tumour core section characterised by inflammatory exudate with infiltration of heterophils (arrows). (c) (i and ii) advance front tumour sections showed virus inclusion bodies with haemorrhage (arrows). (d) (i and ii), the advanced tumour section showed fibrosis (arrows). (H and E: 10X and 40X).

Histopathological examination of the ocular tumour core that replaced the normal eye tissue showed aggregation of neoplastic cells and haemorrhage (Figure 3(a) (i and ii)) in addition to the distribution of inflammatory exudate and infiltration of heterophils (Figure 3(b) (i and ii)). On the other hand, advanced front ocular tumours showed a distribution of intranuclear virus inclusion bodies (Figure 3(c) (i and ii)). Also, in (Figure 3(d) (i and ii)), there is an extensive fibrosis. Taken together, these results indicated that MDV infection enhanced ocular tumours in layer chicken flocks for the first time.

### 3.4. Histopathological Detection of Visceral Lesions Enhanced by Marek's Disease

**Figure 4:**
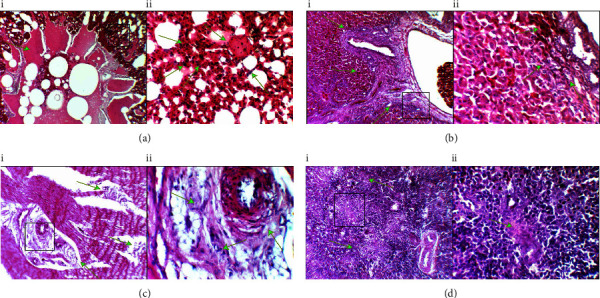
Histopathological sections of the visceral lesions caused by Marek's disease in an infected bird. (a) (i) Lung tumor section characterised by damage with severe atelectasis of lung tissue, the tertiary bronchus filled with pink material smooth to slightly floccular suitable for pulmonary oedema (arrow), and (ii) thickening of the alveolar wall due to aggregation of monomorphic neoplastic cells with an area of necrosis and excessive infiltration of inflammatory cells (arrows). (b) (i and ii) The gizzard section is characterised by dysplasia of the circular muscular layer with aggregation of nuclear inclusion bodies inside the cells (arrows). (c) (i and ii) The liver section showed early tumour formation characterised by monomorphic neoplastic cells and nuclear inclusion bodies with haemorrhage (arrows). (d) (i and ii) A spleen section showed tissue depletion (arrows). (H and E: 10X and 40X).

Further histopathological examination of internal organs has been achieved, and the examination of lung lesions indicated that early tumour formation was characterised by the aggregation of neoplastic cells with extensive infiltration of inflammatory cells, in addition to damage to the lung tissue as well as severe atelectasis with some areas of necrosis and the tertiary bronchus filled with a slightly floccular to smooth pink material susceptible to pulmonary oedema (Figure 4(a) (i and ii)). Similarly, the examination of the liver sections showed early tumour formation characterised by the presence of neoplastic cells accompanied with areas of haemorrhage as well as nuclear inclusion bodies were also observed (Figure 4(b) (i and ii)). While in the gizzard, the results showed dysplasia of the circular muscular layer with aggregation of nuclear inclusion bodies inside the cells (Figure 4(c) (i and ii)). In the spleen, multiple areas of follicular centre depletion were spotted (Figure 4(d) (i and ii)).

## 4. Discussion

Marek's disease (MD) is a widespread lymphoproliferative disease caused by the extremely infectious and oncogenic Herpes virus in poultry [33]. The ocular form of MD is summarised by a change in normal eye colour to grey and an irregular pupil shape, with, in some cases, infiltration of malignant lymphoid cells scattered in different tissue parts of the eye [25, 26, 34]. Histopathological capture revealed good recognition for the eye tissues even with the infiltration of neoplastic lymphoid cells [35, 36]. However, in the current study, a remarkable ocular tumour size has been observed for the first time in Iraq and in the world, replacing the normal eyeball. Microscopically, the invasion of malignant cells induced severe damage to the eye tissues, resulting in the loss of the normal arrangement of the eye tissues and the formation of a very large tumour mass. This may refer to the increased mutagenicity of the MD virus and its ability to enhance aggressive tumour formation.

Vaccination against MD reduces outbreaks successfully. Despite the vastly effective MD vaccines, recently, there has been a growing concern about the evolution of the MD Herpes virus to be more virulent. Subsequently, in the last two decades, significant efforts have been devoted to determine the molecular structure and evolution of the MD causative agent [27–29]. However, vaccination did not block infection or prevent transmission. It is suggested that this may be due to imperfect vaccination or because of the infection with highly virulent strains, as well as virus evolution [37–39]. In addition, it has been reported that vaccine ability is limited against evolving strains of the virus [31, 32]. Consequently, this may explain why such a lesion developed in a vaccinated flock. It has been found that even with the differences in virus strain oncogenicity, the early cytolytic infections have been revealed to be similar. However, very virulent strains exhibit severe oncogenic lesions with a late stage of infection [40, 41]. Interestingly, in the present outbreak, in spite of the vaccination, the chickens showed general clinical signs of MD, with very rapid progress of the ocular tumour in one eye of the infected bird, as well as the early formation of tumours in other organs, as mentioned in the results' section. According to the development of the lesion and histopathological examination, we think that the infection started first in the eye and then spread to the other internal organs.

Furthermore, tumour masses are generally observed in internal organs, namely, the lung, liver, spleen, and heart [19, 33]. The infection with MDV induces the growth of T-cell lymphomas in visceral organs and nerves due to the immunosuppression that accompanies such an infection [42, 43]. It has been known that tumour initiation is hardly observed in vaccinated flocks; therefore, the development of an aggressive form of tumour in vaccinated birds might suggest an evolved strain of the virus [42]. The pioneering discovery of the *meq* gene and its relation to the oncogenicity of the MD virus helped in the detection of virulent strains and the ability to initiate tumours [35, 44]. Although the mutation rate of double-stranded DNA viruses is limited, the *meq* gene mutation rate is much faster than that of other genes [36, 37, 45]. Point mutations in the *meq* gene correlate positively with an increase in the virulence of the MD virus [35, 38, 46, 47]. Hence, our findings suggest that the MD virus has a very aggressive oncogenicity ability and is suitable to invade the internal organs, forming tumours as a first record of such infection in Al-Najaf province/Iraq. The crucial impact of these results may refer to a new feature of Marek's disease. This might be in line with the unique clustering of the DNA sequence of this study from other sequences worldwide and even from those records from Iraq. In previous study, a deletion of 123 bp was noticed in the *meq* gene from samples isolated from Iraq, suggesting an evolutionary signal worth to be revisited in more details in the future studies [48]. While these findings are quite important, the limitation of the present study is that it covered just one geographical region of Iraq. Therefore, this needs to expand the investigation to cover other cities and regions of Iraq.

## 5. Conclusions

To conclude, this study presents a novel confirmed record for an aggressive form of a massive ocular tumour that replaced the whole normal eye tissue caused by Marek's disease virus in a relatively short period (7 weeks). In addition, initial tumour formation has been observed in some internal organs in the vaccinated layer flock. To our knowledge, this is the first record for such an aggressive ocular form caused by MD virus infection in Al-Najaf Province, Iraq, and probably globally. This lesion may be used as a good indication for MD diagnosis if the recurrency increased, which may need further studies and more investigation to study this unusual tumour.

## Data Availability

The data that support the findings of this study are available in the supplementary material of this article.

## Supplementary Materials

Supplementary Materials All files and metadata used in this study are stored in the Open Science Frame domain with project DOI of DOI 10.17605/OSF.IO/4HBZP, please see the following link: https://osf.io/4hbzp/?view_only=d778df15b1da4f6c845835c4459a05ce [49].
