# Supplementary material for: The First Record of an Aggressive Form of Ocular Tumour Enhanced by Marek's Disease Virus Infection in Layer Flock in Al-Najaf, Iraq
Source: Vet Med Int. 2024 Sep 30;2024:1793189. doi: 10.1155/2024/1793189 (PMC11458278; doi:10.1155/2024/1793189)
Supplement: Supplementary Materials — All files and metadata used in this study are stored in the Open Science Frame domain with project DOI of DOI 10.17605/OSF.IO/4HBZP, please see the following link: https://osf.io/4hbzp/?view_only=d778df15b1da4f6c845835c4459a05ce [49]. [file 1793189.f1.docx]

**Supplementary Data Availability**

All files and metadata used in this study are stored in Open Science Frame domain with project DOI of DOI 10.17605/OSF.IO/4HBZP , please see the following link:

<https://osf.io/4hbzp/?view_only=d778df15b1da4f6c845835c4459a05ce> [49]
